# Design and Optimization of a New Anti-reflux Biliary Stent With Retractable Bionic Valve Based on Fluid-Structure Interaction Analysis

**DOI:** 10.3389/fbioe.2022.824207

**Published:** 2022-03-28

**Authors:** Yushan Su, Zhongxia Xiang, Xiaofei Song, Shuxian Zheng, Xinsheng Xu

**Affiliations:** ^1^ Key Laboratory of Mechanism Theory and Equipment Design of Ministry of Education, Tianjin University, Tianjin, China; ^2^ Nankai Hospital, Tianjin, China

**Keywords:** anti-reflux, biliary stent, bionic valve, fluid-structure interaction, optimization

## Abstract

Duodenal biliary reflux has been a challenging common problem which could cause dreadful complications after biliary stent implantation. A novel anti-reflux biliary stent with a retractable bionic valve was proposed according to the concertina motion characteristics of annelids. A 2D equivalent fluid-structure interaction (FSI) model based on the axial section was established to analyze and evaluate the mechanical performances of the anti-reflux biliary stent. Based on this model, four key parameters (initial shear modulus of material, thickness, pitch, and width) were selected to investigate the influence of design parameters on anti-reflux performance via an orthogonal design to optimize the stent. The results of FSI analysis showed that the retrograde closure ratio of the retractable valve primarily depended on initial shear modulus of material (*p* < 0.05) but not mainly depended on the thickness, pitch, and width of the valve (*p* > 0.05). The optimal structure of the valve was finally proposed with a high retrograde closing ratio of 95.89%. The finite element model revealed that the optimized anti-reflux stent possessed improved radial mechanical performance and nearly equal flexibility compared with the ordinary stent without a valve. Both the FSI model and experimental measurement indicated that the newly designed stent had superior anti-reflux performance, effectively preventing the duodenobiliary reflux while enabling the bile to pass smoothly. In addition, the developed 2D equivalent FSI model provides tremendous significance for resolving the fluid-structure coupled problem of evolution solid with large deformation and markedly shortens the calculation time.

## 1 Introduction

Endoscopic biliary stenting is a universal clinical palliative treatment for biliary obstruction (Perri et al., 2013; Pranculis et al., 2017). However, stent placement through the duodenal papilla often damages the function of the Oddi sphincter (Reddy et al., 2006; Hu et al., 2011) and causes the duodenal contents to reflux into the biliary tract (Woods et al., 2005). Biliary infection and cholangitis are common complications (Sung et al., 1992; Okamoto et al., 2006), seriously affecting patients’ quality of life. In previous studies, food fiber refluxed from the duodenum was found in stents, which proved the presence of duodenobiliary reflux (van Berkel et al., 2005; Isayama et al., 2010). Duodenobiliary reflux is a remarkable phenomenon after stent implantation through duodenal papilla and has been considered another critical reason for stent occlusion (Weickert et al., 2001; Misra and Dwivedi 2009).

Choledochojejunostomy and Choledochoduodenostomy are common surgical bypass operations for biliary diseases (Shah et al., 2009). In theory, Choledochojejunostomy can prevent duodenal biliary reflux by keeping food debris away from the bile duct (Dutra et al., 2008). However, Complications such as hemobilia, pancreatitis, bacteremia, biliary leakage, and intraabdominal abscess still often occur after operations (Pitt et al., 1989; Leppard et al., 2011).

Efforts have been made towards the design of anti-reflux biliary stents for preventing duodenobiliary reflux after biliary stent implantation. Recently, some types of anti-reflux stents with different valves have been proposed, such as duckbilled (Yuan et al., 2019), hemispheric (Hu et al., 2011), funnel-shaped (Morita et al., 2018), S-shaped (Lee et al., 2013), nipple-shaped (Hu et al., 2014), wine-glass-shaped (Kim et al., 2013), and windsock-shaped valves (Lee et al., 2016). It has been reported that implantation of anti-reflux biliary stents was safe and feasible, giving its higher technical success rate, longer stent patency, and no influence on the antegrade flow of bile (Dua et al., 2007; Hu et al., 2011; Renno et al., 2019; Yuan et al., 2021). However, it has been still controversial about the validity of anti-reflux stents. For instance, closed valve structures, such as nipple-shaped (Hu et al., 2014) and hemispheric valves (Hu et al., 2011), need to be opened to allow the passage of bile, which increases the outflow resistance of bile. Increasing outflow resistance of bile may also lead to stent migration (Hamada et al., 2019). In comparison, open valve structures such as funnel-shaped (Hamada et al., 2017; Morita et al., 2018) and S-shaped valves (Lee et al., 2013) cannot prevent duodenobiliary reflux completely, even if they have an excellent bile drainage effect. Folding and collapse are the main causes of valve dysfunction and stent occlusion (Leong et al., 2016; Hamada et al., 2019). Bile sludge and food fiber deposited on valves negatively impacted the morphology and function of valves and affected bile flow (Morita et al., 2018). The incidence of adverse events after anti-reflux biliary stent implantation may be as high as 20% (Hamada et al., 2017). In addition, anti-reflux valves such as duckbilled (Yuan et al., 2019), nipple-shaped (Hu et al., 2014), and windsock-shaped valves (Lee et al., 2016) do not significantly improve patient survival. Consequently, ideal anti-reflux biliary stents are still in the stage of design and research.

Finite element analysis (FEA) is a popular method in studying deployment, mechanical behaviors, and fatigue performance of stents (Jovicic et al., 2014; Qiao and Zhang 2014; Bobel and McHugh 2018; Wei et al., 2019; Yaakobovich et al., 2020; Xue et al., 2021). Establishing a fluid-structure interaction (FSI) model is an excellent method to investigate the anti-reflux performance of the valve because it can reflect the dynamic changes of the structural and fluid domains. Nevertheless, the conventional 3D FSI analysis for the complex models with large deformation would take a very long time, even several months.

Therefore, this article aimed to propose a novel anti-reflux biliary stent with a retractable bionic valve and develop a 2D equivalent FSI model to assess the mechanical performances of the anti-reflux biliary stent. Based on this model, initial shear modulus of material, thickness, pitch, and width was investigated as a function of the relevant parameters of anti-reflux performance using an orthogonal design method. Finally, the anti-reflux biliary stent with an optimized retractable valve was proposed.

## 2 Structure of Anti-reflux Biliary Stent With a Retractable Bionic Valve

**FIGURE 1:**
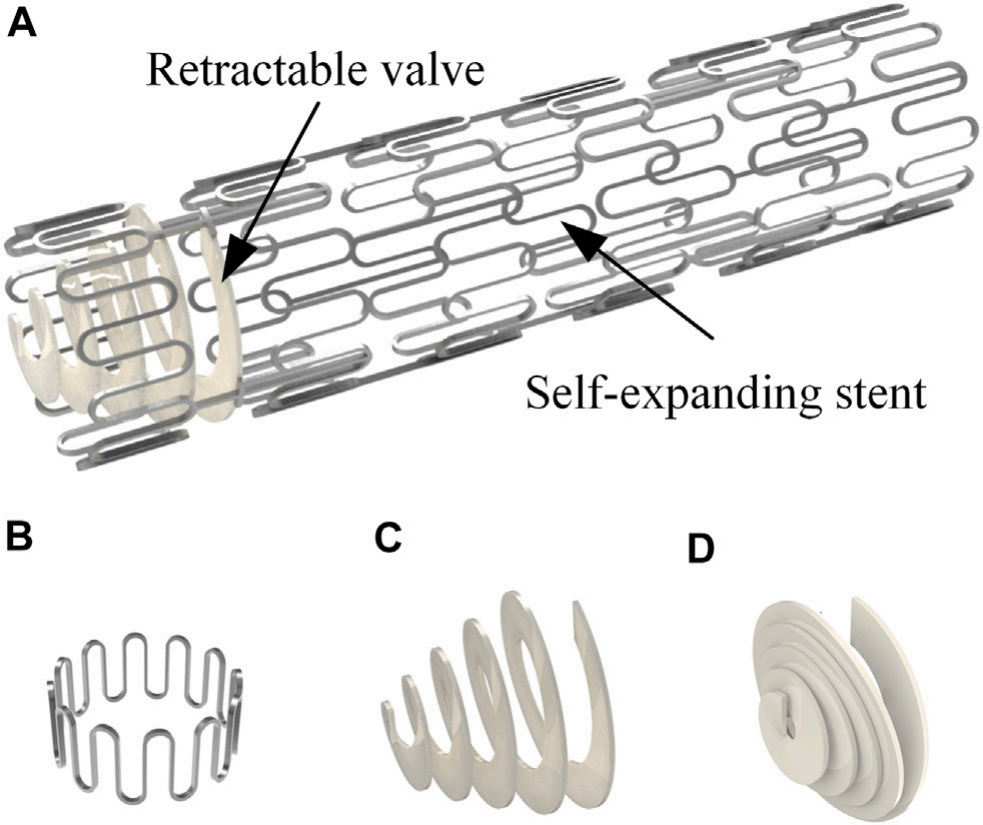
**(A)** Anti-reflux biliary stent with a retractable valve; **(B)** single unit of the stent; valve status in **(C)** antegrade flow of bile and **(D)** duodenal contents reflux.

**FIGURE 2:**
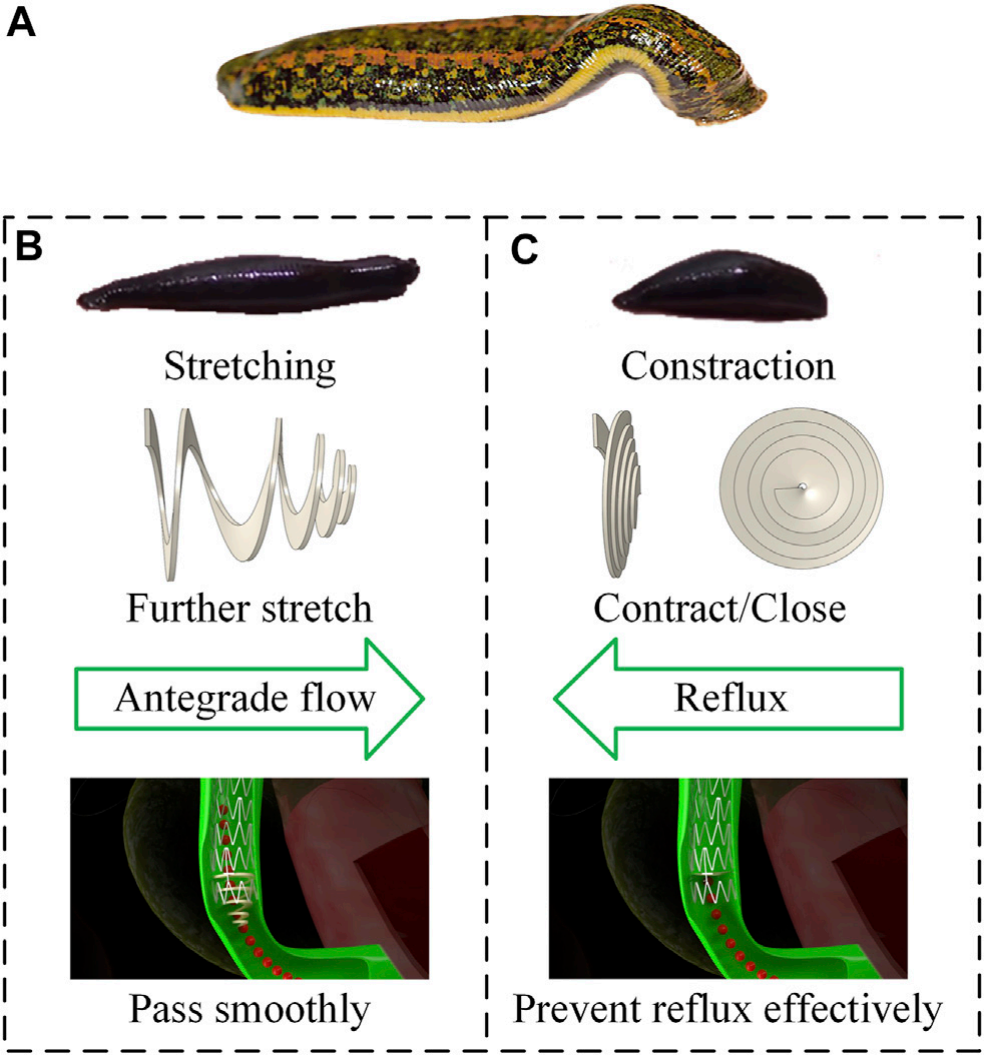
**(A)** Morphology of leech; design principle for **(B)** antegrade flow **(C)** duodenal biliary reflux.

Figure 1A illustrates the structure of a novel anti-reflux biliary stent with a retractable valve. The nitinol self-expanding stent comprised a series of parallel arc struts in circumferential rows (Figure 1B) connected by bridges. The 10 mm external diameter and 9.4 mm internal diameter was used in this work. The valve was made of hyperelastic material and attached to the duodenal end of the stent. This retractable cone spiral valve was designed according to the concertina motion characteristics of annelids, such as leech (Figure 2A), to achieve the stretching and contraction under the pressure of the antegrade and retrograde flow. When bile duct pressure increased, the retractable valve further stretched to ensure the antegrade flow of bile, as shown in Figure 1C and Figure 2B; When duodenal pressure rosed, the valve contracted and closed to prevent duodenal reflux, as shown in Figure 1D and Figure 2C.

## 3 2D Equivalent Fluid-Structure Interaction Model

**FIGURE 3:**
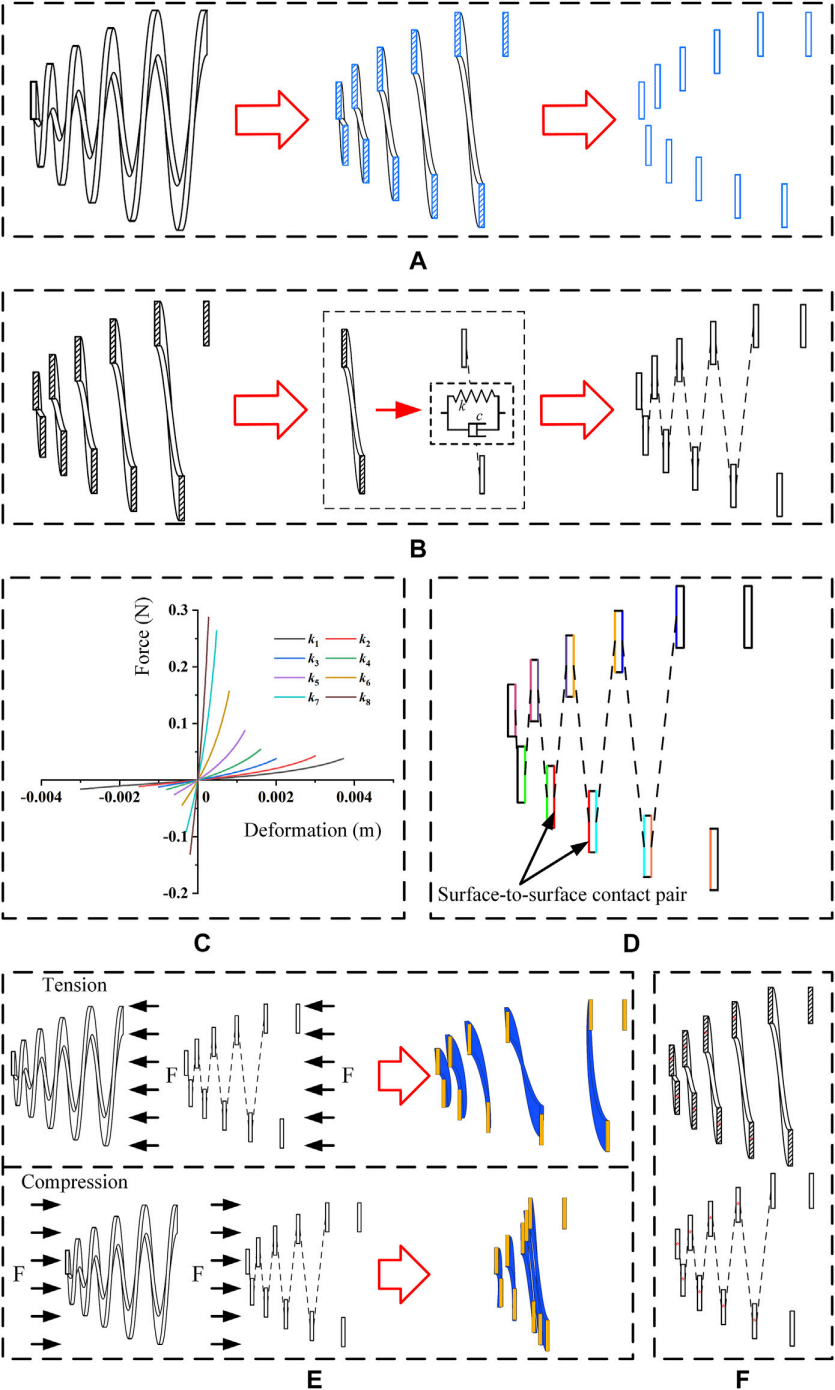
**(A)** Extraction of the maximum axis section; **(B)** adding axial nonlinear spring connections between sections; **(C)** initial spring stiffness value of each axial nonlinear spring connection; **(D)** surface-to-surface contact pairs in the 2D model (a color represents a pair); **(E)** deformation fitting of the 3D and 2D models (blue represents 3D model, orange represents 2D equivalent model) and **(F)** fitting points in 3D and 2D models (* represents a fitting point).

The objective of this model was to estimate the mechanical performances of the new anti-reflux biliary stent. Based on the maximum axis section of the 3D model of a valve, a 2D model with similar mechanical properties was established by adding nonlinear connection attributes between sections. Figure 3A illustrates the extraction of the maximum axis section of the 3D model. The geometrical models were generated using Solidworks 2017 (Dassault Systemes, France) and were transformed into FEM code Abaqus 2018 (Dassault Systemes, France) for analysis. The 3D model was modeled as hyperelastic materials using Mooney-Rivlin (MR) model, and the strain energy density *W* can be written as (Kim et al., 2012)

$$W=C_{10}\left(\bar{I_{1}}-3\right)+C_{01}\left(\bar{I_{2}}-3\right)$$ (1)

$$\mu_{0}=2\left(C_{10}+C_{01}\right)$$ (2)

where *C*_10_ and *C*_01_ are empirical constants for the material, $\bar{I_{1}}$ is the first deviatoric strain invariant, $\bar{I_{2}}\;$ is the second deviatoric strain invariant, and *μ*_0_ is the initial shear modulus of the material. Here, *C*_10_ = 152.5 MPa and *C*_01_ = 19.6 MPa, which was adopted from previous research (Avanzini and Battini 2016). The mechanical properties of the 2D model material are: Mass density *ρ* = 7,800 kg/m^3^ and Young’s modulus *E* = 210 GPa. The axial deformation of the 3D valve has the most significant impact on the anti-reflux performance. Therefore, in the establishment of the 2D model, the torsional deformation of sections was ignored; only the axial deformation was considered. Due to the cone spiral structure, the 3D model deforms nonlinearly under tensile and compressive loads. In order to imitate the axial deformation of the 3D model, an axial nonlinear spring connection *k* was added between the sections corresponding to each half-circle valve in the 2D model, as shown in Figure 3B. In addition, a parallel linear damping *c* equal to 0.1 was added in the connection to ensure the stable deformation of the 2D model, shown in Figure 3B. For easy adding the connection, a reference point was established at the midpoint of the axial side of each section. Then the reference point was coupled with its side in the 2D model. The axial nonlinear spring connection was established between the reference points of the sections corresponding to each half-circle valve. Due to the difference in axial mechanical properties of each half-circle valve, eight axial nonlinear spring connections with different spring stiffness were established in the 2D model. The connections were named *k*_1_ to *k*_8_ successively, from the large end to the small end of the valve. The initial spring stiffness value in each connection was obtained by axial tensile and compressive simulation on the corresponding half-circle valve. In the mechanical simulation of each half-circle valve, one end of the half-circle valve was fixed, and the other end was applied with appropriate axial displacement load. The displacement load was along the positive direction of the axis in the tensile simulation and the negative direction of the axis in the compressive simulation. The axial force versus axial displacement of each half-circle valve was obtained according to the mechanical simulation, shown in Figure 3C. Each curve represents the initial value of spring stiffness in the corresponding correction. A static general step was set for analysis of the 3D and 2D models, in which the initial increment size was 5e-5. Self-contact of the 3D model occurs in compressive simulation. Therefore, the Penalty model modeled the frictional behavior with a friction coefficient equal to 0.2 for self-contact of the 3D model. Similarly, the adjacent sections of the 2D model contact in the compressive simulation. Surface-to-surface contact pairs between adjacent sections of the 2D model were set, and the friction coefficient was 0.2, shown in Figure 3D. The same color represents the same contact pair in the 2D model. The first ring of the large end of the valve was fixed with the stent. This part was constrained in all directions, both in the 3D model and 2D model. Since only the axial deformation of sections was considered in the establishment of the 2D model, the other sections of the 2D model were constrained to move only in the axial direction. The 3D model was meshed with 263,704 linear hexahedral elements (C3D8RH), and the 2D model was meshed with 1464 linear quadrilateral elements (CPS4I). In order to match the mechanical properties of the 2D model and 3D model, it is necessary to carry out a mechanical simulation on 2D and 3D models under appropriate load. Figure 3E shows that the axial load equal to 1000 Pa was applied to the 3D model and each section of the 2D model, respectively, for tensile and compressive analysis. The center point of each section in 2D and 3D models was the fitting point, as shown in Figure 3F. The axial coordinates of these points were measured in the deformed 3D and 2D models. According to the axial coordinate error of each fitting point, the spring stiffness in the eight nonlinear spring connections was adjusted successively until the axial coordinate errors were less than 10% in tension and compression deformation.

**FIGURE 4:**
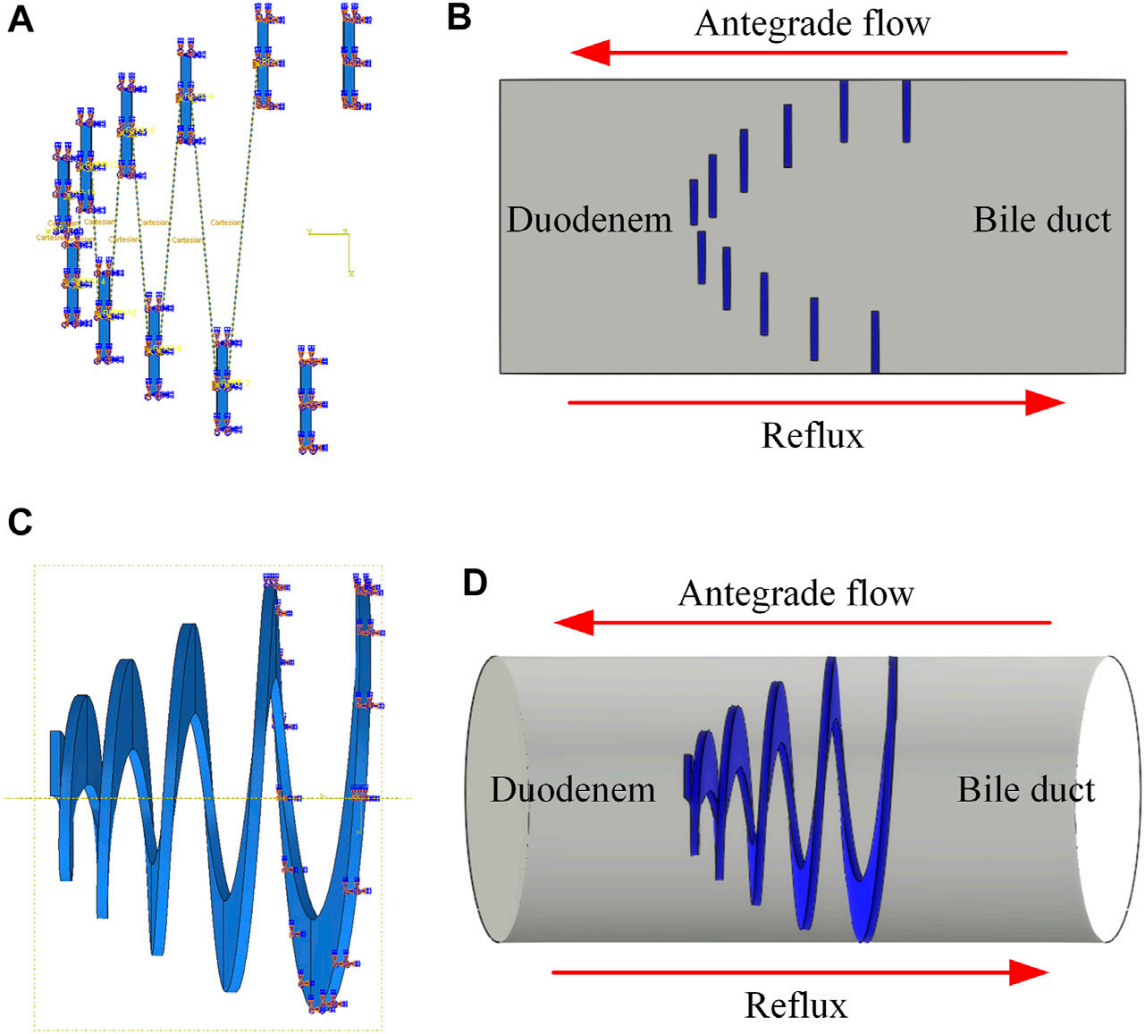
**(A)** Structural domain and **(B)** fluid domain of 2D equivalent FSI model; **(C)** Structural domain and **(D)** fluid domain of 3D FSI model.

Based on the above 2D model, a 2D equivalent FSI model was developed, and the anti-reflux performance was analyzed via commercial software Abaqus 2018 and XFlow 2018 (Next Limit Technologies, Spain). The structural domain of the model was created in Abaqus 2018. A dynamic implicit step was set, and the simulation time was 0.3 s. The material properties, frictional behavior, boundary conditions, and mesh elements were consistent with the analysis of the 2D model. In addition, a Fluid-Structure Co-simulation boundary interaction was created in the mesh form of a 2D model to establish the information transmission between fluid domain and structural deformation. After that, a geometrical mesh model was exported from Abaqus 2018 and imported into XFlow 2018. A 2D single-phase internal engine was created, and Abaqus structural analysis was set. A rectangular fluid domain was created, and the pressure difference between the bile duct and duodenum was considered as the boundary condition. It has been reported that the duodenal biliary reflux occurred in phase Ⅲ of duodenal interdigestive cycle activity (Worthley et al., 1989). The reported amplitude ranged from 25.6 to 47.7 mmHg (Andersen et al., 1991), equivalent to the pressure between 3.14 and 6.36 kPa. It was also reported that common bile duct pressure was 1.12 kPa (Kuchumov 2018). Therefore, when duodenal biliary reflux occurs, the retrograde pressure (the pressure difference between inlet and outlet) ranges from 2.29 to 5.24 kPa. The lowest pressure was chosen as the boundary condition, which the inlet pressure was set to 2.29 kPa and the outlet pressure was 0 Pa. Water, with similar physical properties to human healthy bile (Al-Atabi et al., 2010), was adopted as the fluid domain for simplifying the simulation. The simulation time in XFlow was consistent with 0.3 s in Abaqus. Fixed custom time step mode was set, and resolved scale was 0.0001 m. For comparison, a 3D FSI model was also established. A 3D single-phase internal engine was created, and a cylindrical fluid domain was created in XFlow. The material properties, frictional behavior, boundary conditions, and mesh elements were consistent with the static analysis of the 3D model, and the other steps were the same as the establishment of the 2D equivalent FSI model. Figure 4 shows the structural domain and fluid domain of the 2D equivalent FSI model and the 3D FSI model.

**FIGURE 5:**
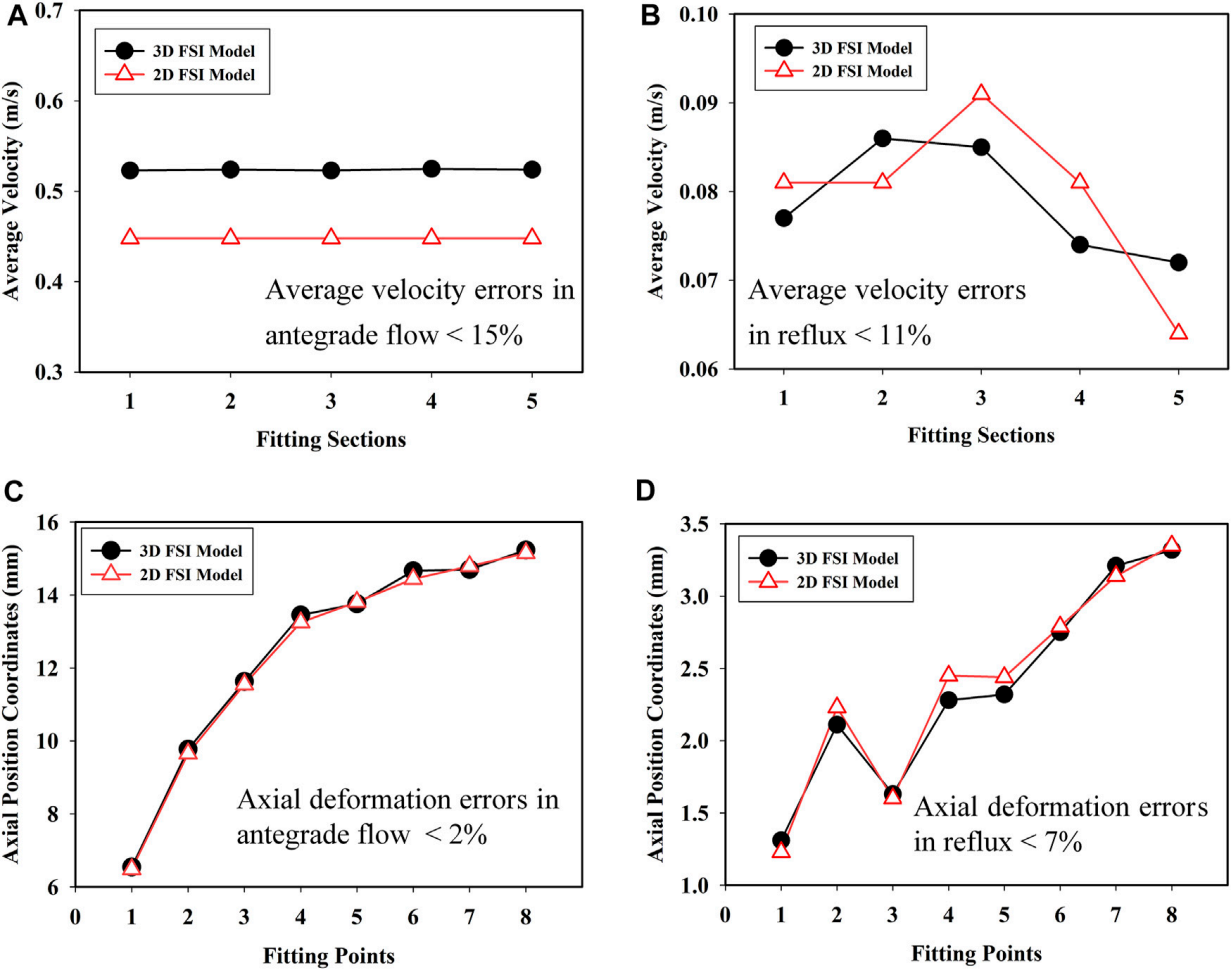
Average velocities in **(A)** antegrade flow of bile and **(B)** reflux; axial position coordinates in **(C)** antegrade flow of bile and **(D)** reflux for the 3D and 2D FSI models.

The average velocities of flow in 3D and 2D models were calculated under the same conditions, respectively. The flow (fluid-passing volume per unit time) was obtained based on the simulation results from the 3D and 2D models. In each fluid domain, five sections were evenly selected along the axial direction of the valve. The average velocities for the 3D and 2D models in antegrade flow and reflux are shown in Figures 5A,B. The results show that the average velocity errors between 3D and 2D models for antegrade flow and reflux were less than 15 and 11%, respectively. In addition, the axial coordinates of the center points of each section in the deformed 3D and 2D models were measured, shown in Figures 5C,D. The axial deformation error of the 3D FSI model and 2D FSI model in antegrade flow was less than 2% and was less than 7% in reflux. The model fitting is proved to be satisfying. Therefore, the 2D FSI model could reflect the performance of the 3D model. It was feasible to apply the 2D FSI model to simplify the fluid-structure coupled problem of evolution solid with large deformation. The simulation of 2D FSI model only took a few days, which was only 1/10 of that of the ordinary 3D FSI model.

## 4 Structure Optimization and Performance Evaluation

### 4.1 Optimal Design of Valve

Orthogonal design is an efficient and economic research method for multi-factor and multi-level analysis. Based on the establishment of 2D equivalent FSI model, an orthogonal design with four factors and three levels was designed to investigate the effect of initial shear modulus of material, thickness, pitch, and width on anti-reflux performance and optimize design parameters of the retractable valve. The orthogonal factors and levels were illustrated in Table 1 *L*_9_ (3^4^) orthogonal table was chosen, as shown in Table 2.

**TABLE 1:** Factors and levels of orthogonal design.

Factors	Parameters	Level 1	Level 2	Level 3
A	Initial Shear Modulus (MPa)	688.4	172.1	344.2
B	Thickness (mm)	0.275	0.25	0.225
C	Maximum Pitch (mm)	2.0	1.9	2.1
D	Width (mm)	2.1	2.0	1.9

**TABLE 2:** *L*_*9*_ (3^4^) Orthogonal design array.

No	Factor A	Factor B	Factor C	Factor D
1	1	1	1	1
2	1	2	2	2
3	1	3	3	3
4	2	1	2	3
5	2	2	3	1
6	2	3	1	2
7	3	1	3	2
8	3	2	1	3
9	3	3	2	1

The hyperelastic properties of rubber are one of the reasons for the large deformation of the retractable valve. In this paper, the initial shear modulus *μ*_0_ was used to express the hyperelastic properties of the material. *μ*_0_ = 688.4 MPa corresponded to *C*_10_ = 305 MPa and *C*_01_ = 39.2 MPa; *μ*_0_ = 172.1 MPa corresponded to *C*_10_ = 76.25 MPa and *C*_01_ = 9.8 MPa; *μ*_0_ = 344.2 MPa corresponded to *C*_10_ = 152.5 MPa and *C*_01_ = 19.6 MPa. The pitch of the retractable valve was a decreasing arithmetic sequence with a tolerance of 0.4 mm from the large end to the small end, which could be calculated according to the maximum pitch. The width of the first four circles from the large end to the small end of the valve was a fixed value, while the width of the fifth circle was a gradual amount to ensure the same overlap rate between every two circles. The overlap ratio *δ* of each layer is calculated as follows:

$$\delta =\left(1-\frac{\Delta d}{2L}\right)\times 100\%$$ (3)

where *∆d* is the difference between the outer diameter of each layer, and *L* is the width of the valve. In particular, the width mentioned later referred to the value except for the fifth circle.

A set of 2D equivalent FSI models was established to analyze anti-reflux performances. The retrograde closure ratio was regarded as an evaluation index. The retrograde closure ratio is the ratio of the retrograde compression displacement of the valve in FEA and the ultimate value of the retrograde compression displacement of the model. The ultimate value *X* can be calculated by the following expression:

X=H−p−5t (4)

where *H* is the initial height, *p* is the maximum pitch, and *t* is the thickness of the valve.

### 4.2 Statistical Analysis

Multi-way factorial analysis of variance (ANOVA) F-test at a 5% significance level was applied to examine the influence of hyperelastic properties of the material, thickness, pitch, and width on retrograde closure ratio.

### 4.3 Performance Evaluation of the Optimized Anti-reflux Biliary Stent

The anti-reflux performance of the optimized retractable valve was evaluated by a 3D FSI model. The structural domain of the model was created in Abaqus 2018, and the fluid domain was created in Xflow 2018. The material properties were determined according to the optimization results. The other steps were the same as the above.

Radial mechanical performances and flexibility of the optimized anti-reflux biliary stent were appraised via Abaqus 2018 (Ribeiro et al., 2018). Two rings of stent were considered in radial compression, and the entire length of stent was considered bending. The material properties of the stent were shown in Table 3 (Azaouzi et al., 2012). The Penalty model modeled the frictional behavior with a friction coefficient equal to 0.2 for self-contact of the stent and all surface-to-surface contact pairs of stent and valve. The stent was meshed with 43184 linear hexahedral elements (C3D8I), and the valve was meshed with 19290 linear hexahedral elements (C3D8H). Radial pressure was applied to the outer surface in radial compression, and an angular displacement was applied at both ends of the stent in bending.

**TABLE 3:** Parameters of Nitinol material.

Parameters	Value
Density	6450 Kg/m^3^
Austenite elasticity	68000 MPa
Austenite Poisson’s ratio	0.3
Martensite elasticity	36000 MPa
Martensite Poisson’s ratio	0.3
Transformation strain	0.04
(δσ/δT) loading	6.5
Start of transformation loading	520 MPa
End of transformation loading	635 MPa
Reference temperature	37°C
(δσ/δT) unloading	6.5
Start of transformation unloading	180 MPa
End of transformation unloading	26 MPa
Start of transformation stress during loading in compression	780 MPa

## 5 Results

### 5.1 Retrograde Closure Ratio

**FIGURE 6:**
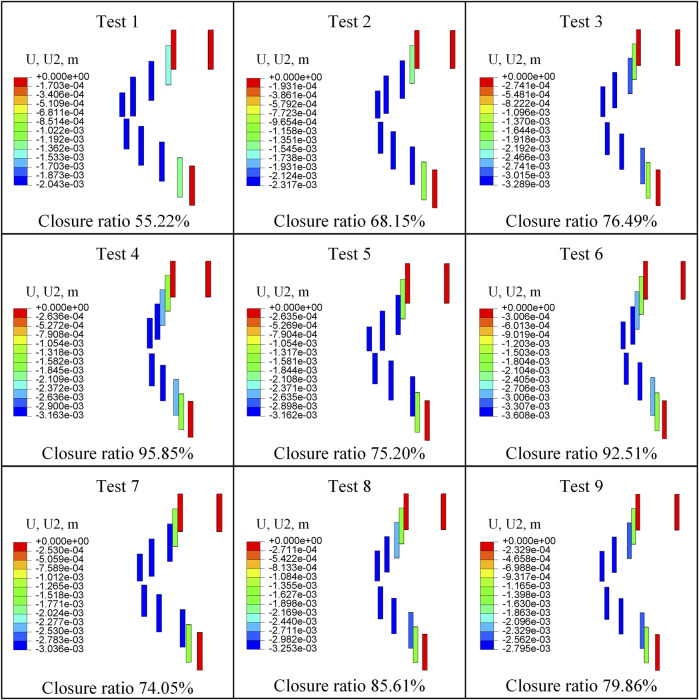
Retrograde compressional deformation and closure ratio of Test 1-9.

Figure 6 shows the retrograde compressional deformation and closure ratio of nine tests. The minimum retrograde closure ratio was 55.216%, and the maximum retrograde closure ratio was 95.848% in all nine tests.

**FIGURE 7:**
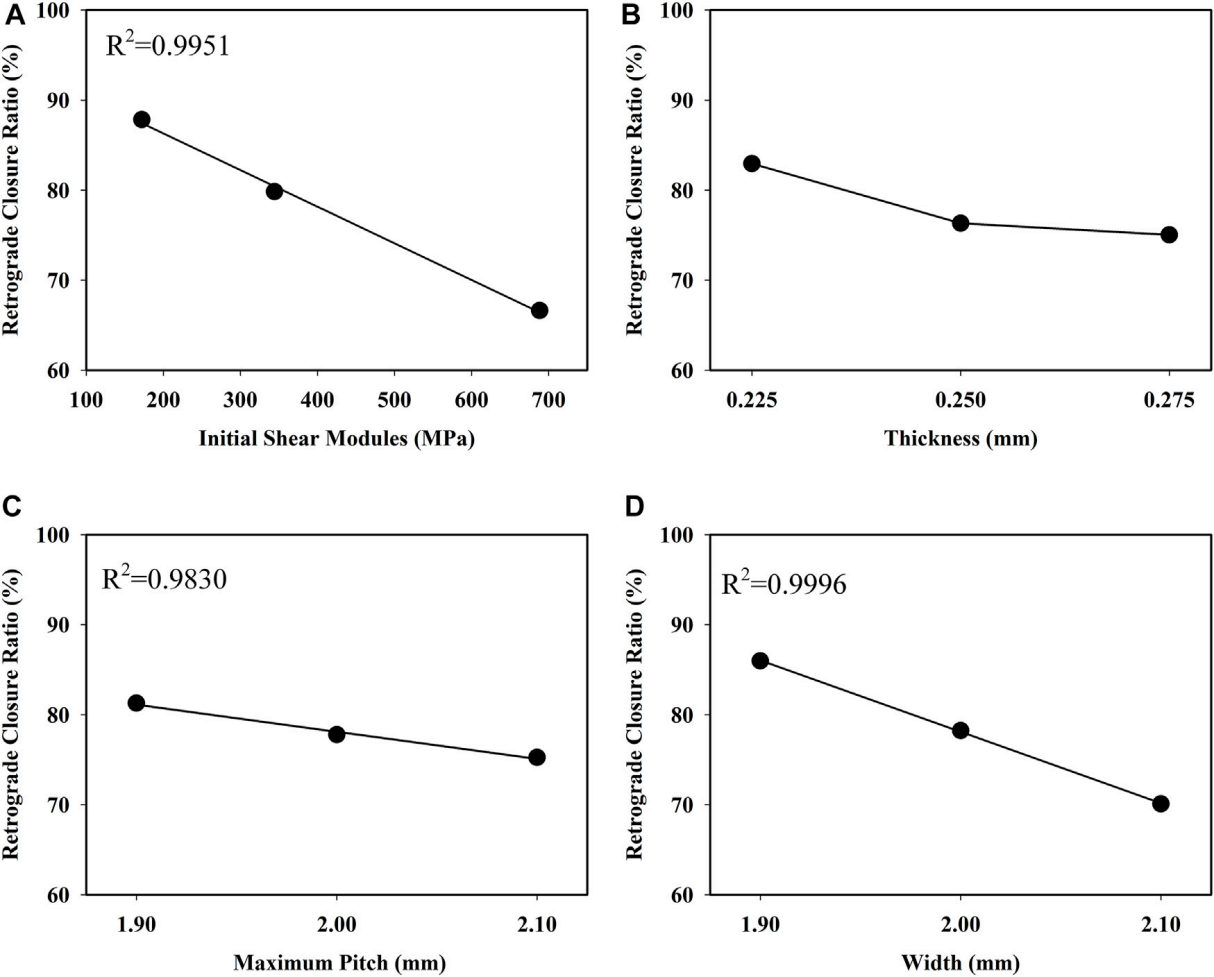
Influence of **(A)** initial shear modules, **(B)** thickness **(C)** pitch, and **(D)** width on retrograde closure ratio of the retractable valve.

The retrograde closure ratio as a function of the initial shear modulus of the material is plotted in Figure 7A. This demonstrated that the retrograde closure ratio of valve significantly decreased linear with increasing the initial shear modulus of material with a coefficient of determination *R* ^*2*^ = 0.9951. With the initial shear modulus increase from 172.1 to 688.4 MPa, the retrograde closure ratio decreased by about 24%, from 87.85 to 66.62% (*p* < 0.05).

The retrograde closure ratio as a function of the thickness is shown in Figure 7B. The retrograde closure ratio decreased slightly from 82.95 to 75.04%, with the increase of thickness from 0.225 to 0.275 mm (*p* > 0.05).

The retrograde closure ratio as a function of the pitch is given in Figure 7C. It showed that the retrograde closure ratio had a small linear decrease with the pitch increase with a coefficient of determination *R* ^*2*^ = 0.9830. The change in pitch was not prominent as a function of the retrograde closure ratio (*p* > 0.05).

The retrograde closure ratio as a function of the width is illustrated in Figure 7D. It indicated that the retrograde closure ratio decreased linear by increasing the width with a coefficient of *R* ^*2*^ = 0.9996. The influence of the width on the retrograde closure ratio was not essentially (*p* > 0.05). At the width of 1.9 mm, the retrograde closure ratio was 85.98%. While increasing the width to 2.1 mm, the retrograde closure ratio decreased to 70.09%.

### 5.2 Optimization and Performance Evaluation

According to the retrograde closure ratio, the design parameters of the retractable valve were optimized, and the parameters with the maximum level value of the performance indicators were selected. The initial shear modulus of the material was 172.1 MPa, the thickness was 0.225 mm, the maximum pitch was 1.9 mm, and the width was 1.9 mm. The initial height of the optimized valve was 6.525 mm. Based on the numerical simulation with this set of optimized designed parameters, the retrograde closure ratio reached about 95.89% when the retrograde pressure was 2.29 kPa.

**FIGURE 8:**
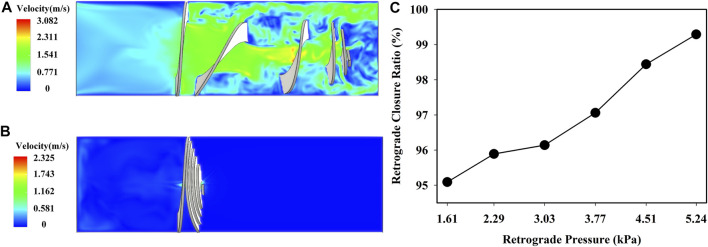
Fluid velocity field of the optimized valve in **(A)** antegrade flow of bile and **(B)** duodenal biliary reflux at *p* = 2.29 kPa; **(C)** retrograde closure ratio versus retrograde pressure.

Figures 8A,B show the deformation of the optimized valve when the pressure was 2.29 kPa. The valve stretched and opened to provide sufficient flow space for antegrade flow, and the height of the deformed valve was about 11 mm. Furthermore, the valve was compressed to prevent the retrograde flow, and the outlet mass flow was almost zero. The height of the optimized valve after closure was about 2.6 mm. The retrograde closure ratio as a function of the retrograde pressure is shown in Figure 8C. The retrograde closure ratio of the optimized valve was increased with increasing the retrograde pressure. At the retrograde pressure of 1.61 kPa, the retrograde closure ratio was 95.09%. While increasing the pressure to 5.24 kPa, the retrograde closure ratio increased to 99.29%. It indicated that the optimized valve could effectively prevent duodenobiliary reflux.

**FIGURE 9:**
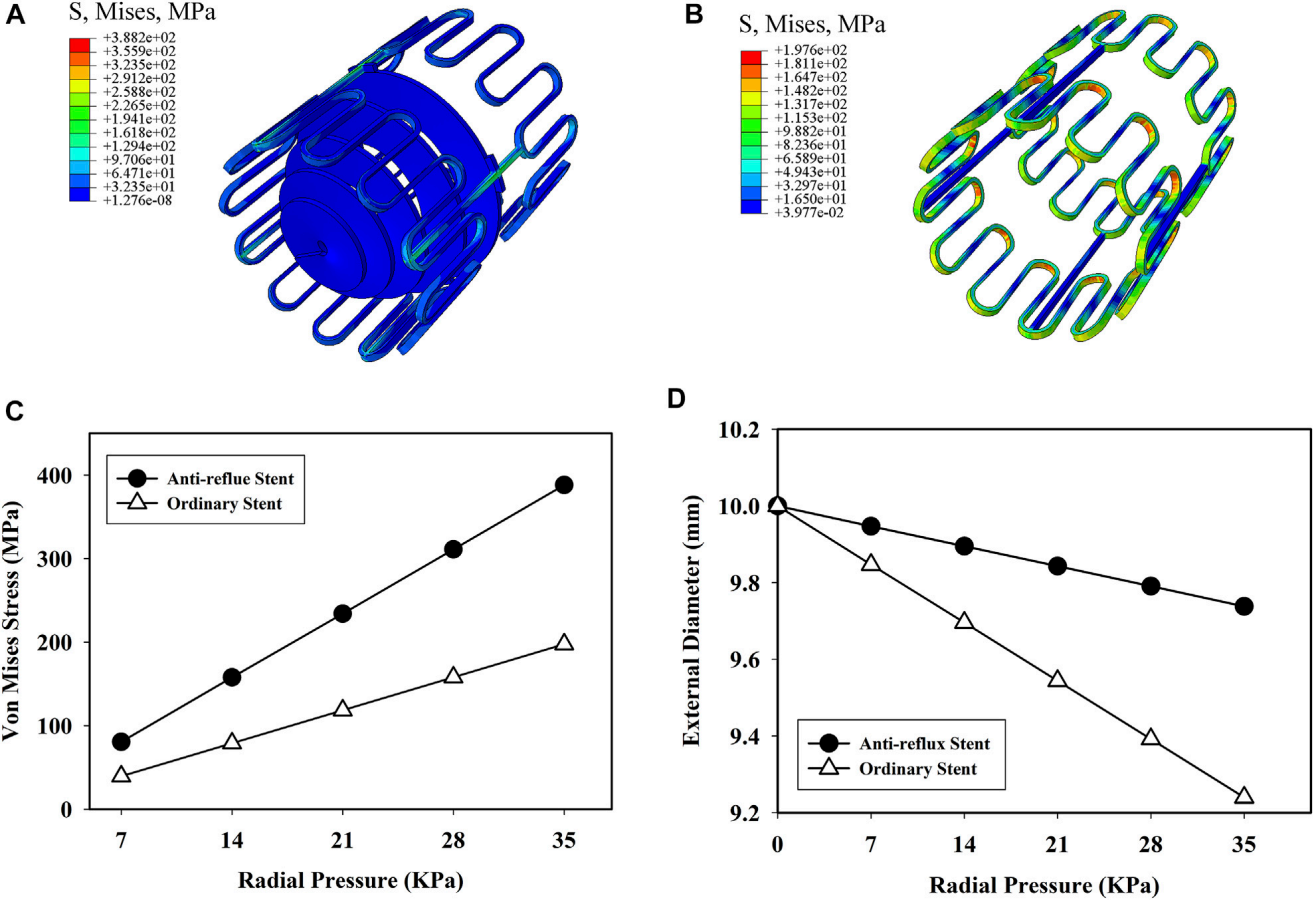
Von mises stress distribution of **(A)** anti-reflux stent and **(B)** ordinary stent at *p* = 35 kPa; **(C)** von mises stress and **(D)** external diameter of the anti-reflux stent and ordinary stent versus radial pressure.

**FIGURE 10:**
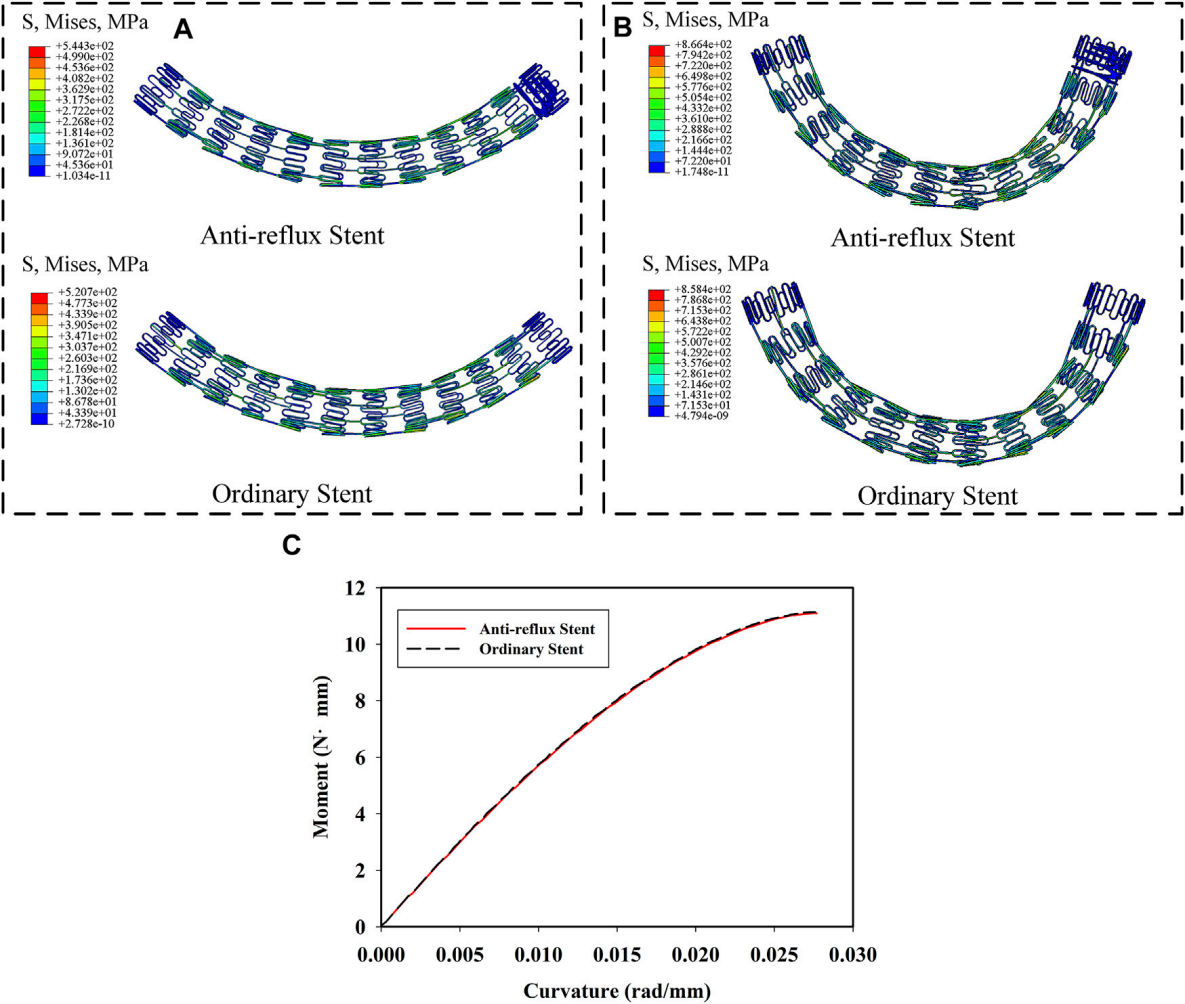
Von mises stress distribution of anti-reflux stent and ordinary stent at **(A)** α = 40° and **(B)** α = 70°; **(C)** moment of the anti-reflux stent and ordinary stent versus curvature.

Figures 9A,B show the distributions of the von Mises stress when the radial pressure was 35 kPa. High stress was located at the bridge and struct connections of the anti-reflux stent, while at the struts of the ordinary stent. The stress of the anti-reflux stent was about twice that of the ordinary stent, and the radial deformation of the anti-reflux stent was less than that of the stent under the same pressure, as shown in Figures 9C,D, indicating that the radial support stiffness of the stent at the connection with valve increased. Figures 10A,B show the distributions of the von Mises stress when the bending angle was $40^{{}^{\circ}}$ and $70^{{}^{\circ}}$ . The anti-reflux stent had similar bending deformation characteristics to the ordinary stent. As shown in Figure 10C, the bending stiffness of the two stents was similar, indicating that the valve will not adversely affect the flexibility of the stent.

## 6 *In vitro* Experimental Verification

**FIGURE 11:**
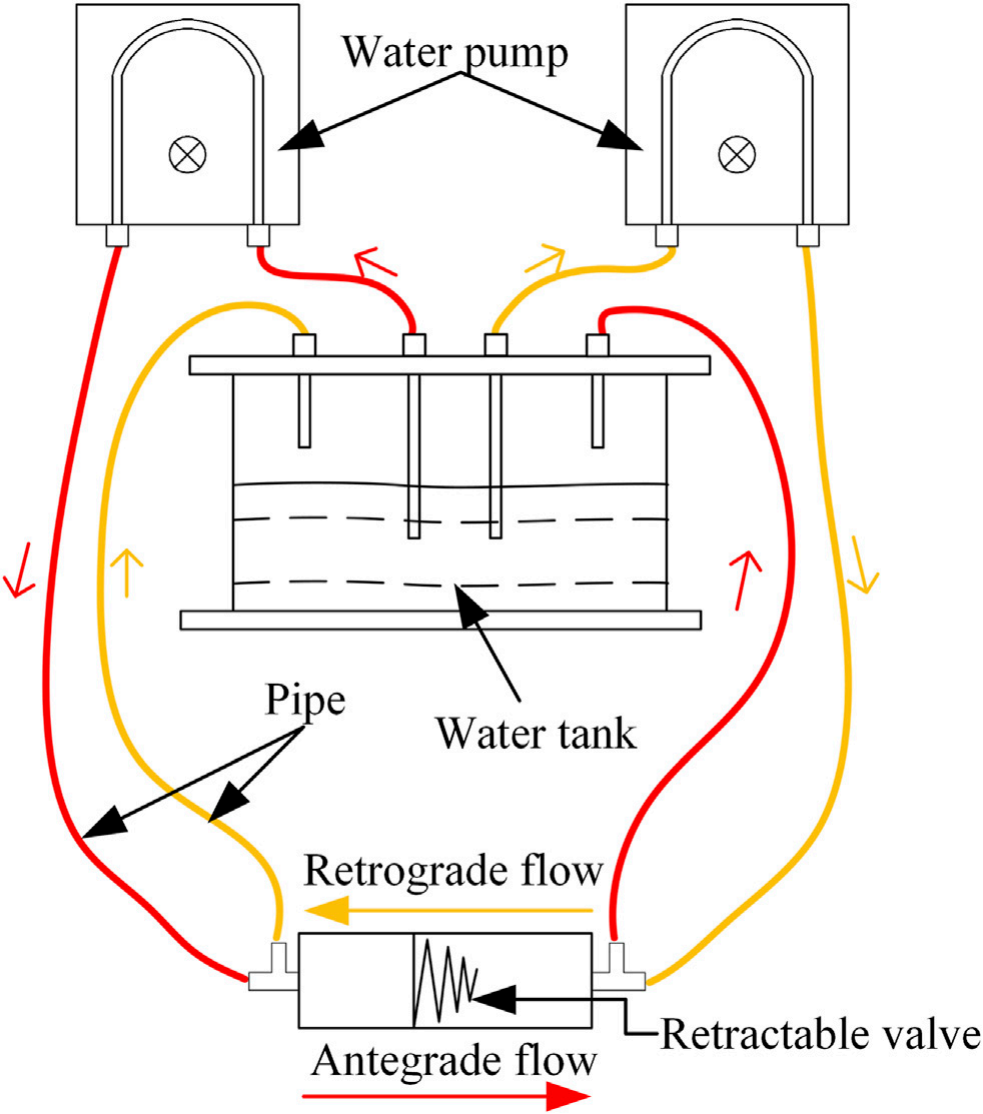
Diagram of *in vitro* experiment of fluid properties (red represents an antegrade flow of water, orange represents a retrograde flow of water).

The effect of the valve on antegrade flow and reflux was measured with an enlarged model. The diagram of the experiment is shown in Figure 11. Two water pumps (HJ-4500; Shanghai Dayouu Instrument; China) were water circulation power sources. The antegrade flow of water passed through the large end of the valve to the small end, and water refluxed from the small end of the valve to the large end. Five groups of experiments with different pressure of flow were carried out. The flow (fluid-passing volume per unit time) in the pipeline with the anti-reflux valve and the flow in the pipeline without the anti-reflux valve was measured three times for each group, and the fluid passing ratio was calculated. The fluid passing ratio is the ratio of the flow in the pipeline with the anti-reflux valve to the flow in the pipeline without the anti-reflux valve. In addition, the compression displacement of the valve was measured three times for each group, and the retrograde closure ratio was calculated. The retrograde closure ratio is the ratio of the retrograde compression displacement of the valve in the experiments to the ultimate value of the retrograde compression displacement of the valve. The calculation of ultimate value is consistent with that in FEA.

**FIGURE 12:**
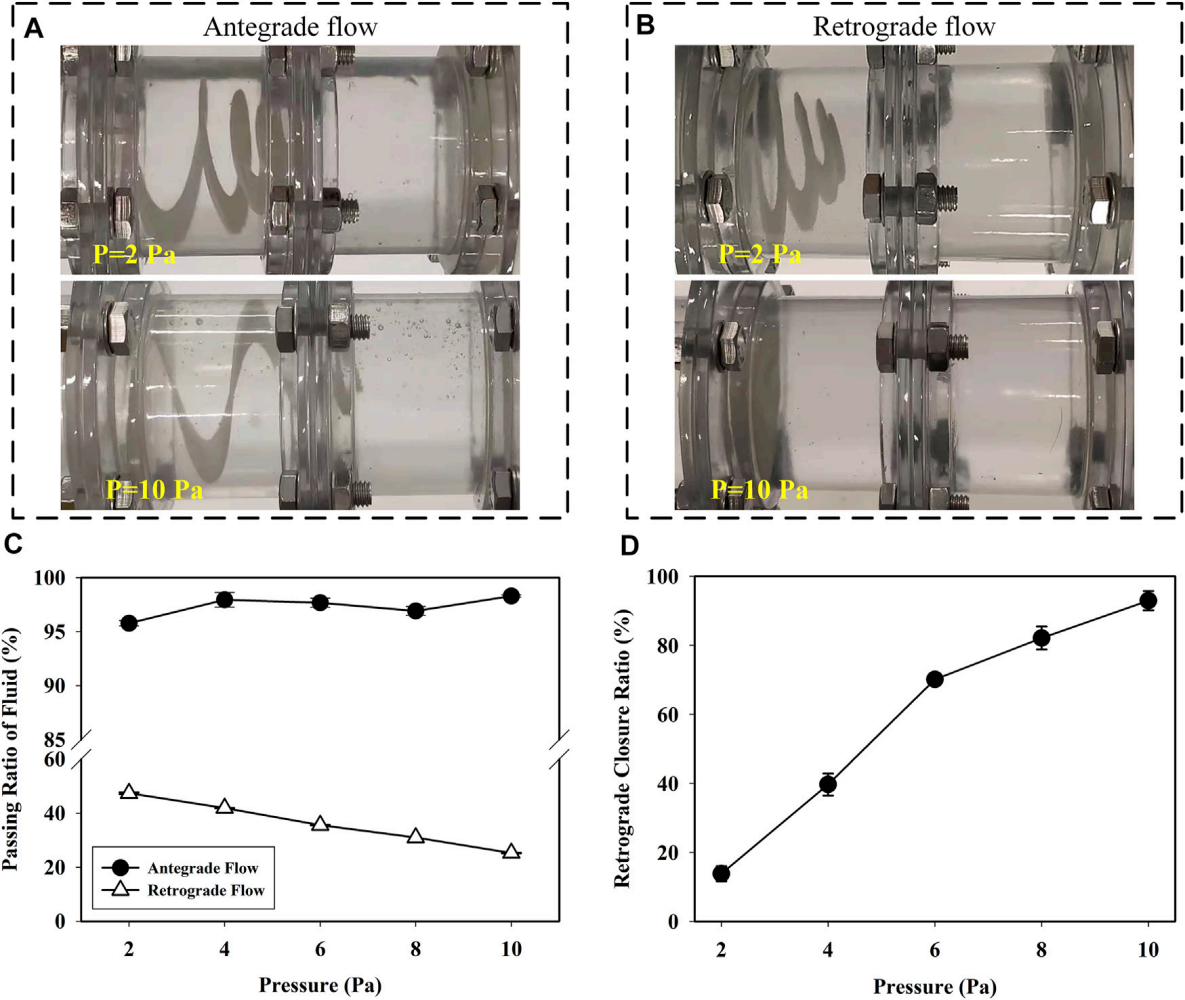
Status of the valve in **(A)** antegrade and **(B)** retrograde flow at pressure was 2 and 10 Pa; **(C)** the passing ratio of antegrade and retrograde flow versus pressure in the experiment and **(D)** the retrograde closure ratio of valve versus pressure in the experiment.

As shown in Figures 12A,B, the retractable valve remained open in antegrade flow and closed with the increase of retrograde pressure. Figure 12C reveals the passing ratio in antegrade and retrograde flow as a function of pressure in the experiment. The passing ratio was higher than 95% in antegrade flow, indicating that the retractable valve did not affect the passage of antegrade fluid. The passing ratio decreased obviously with the increase of retrograde pressure (*p* < 0.001). The increase of pressure from 2 to 10 Pa resulted in decreasing the passing ratio from 47.41 to 25.25%. The retrograde closure ratio of the valve as a function of the pressure is shown in Figure 12D. The retrograde closure ratio was increased with the increasing pressure. At the pressure of 2 Pa, the retrograde closure ratio was 13.82%. While increasing the pressure to 10 Pa, the retrograde closure ratio increased to 92.95%. The results indicated that the retractable valve could contract and close to prevent the retrograde fluid passage effectively.

## 7 Discussion

This study proposed a novel anti-reflux biliary stent with a retractable bionic valve, and a 2D equivalent FSI model was developed to estimate the mechanical performances of the anti-reflux biliary stent. Based on this model, an orthogonal design was designed to investigate the influence of hyperelasticity of material, thickness, pitch, and width on the retrograde closure ratio of the retractable valve. Finally, an anti-reflux stent with the optimized retractable valve was determined.

The 2D equivalent FSI model dramatically reduces the simulation time of a complex model with large deformation. Compared with the ordinary 3D FSI model, the 2D equivalent FSI model simulation only took a few days, saving 90% of simulation time. It is of great significance to simplify the FSI calculation for parametric analysis of solid with large deformation.

The ANOVA confirms that the initial shear modulus of material mainly improves the retrograde closure ratio of the retractable valve, but thickness, pitch, and width is irrelevant to the retrograde closure ratio. In other words, the initial shear modulus of material is the main factor affecting the stiffness of the valve. Therefore, it is feasible to design a retractable valve with multiple materials to optimize the stiffness of the valve for the improvement of anti-reflux performance in the future.

There are many advantages of the newly designed anti-reflux biliary stent with a retractable bionic valve. Both FEA and experimental results show that the retractable valve stretches and opens to ensure the antegrade flow, as shown in Figure 8A, 12A. In addition, the valve contracts and closes to prevent the passage of flow when the pressure of retrograde flow rises. With the increase of retrograde pressure, the optimized valve is almost completely closed. The pressure used in the experiment was far less than the actual pressure of duodenal biliary reflux (Andersen et al., 1991; Sun et al., 2011). The passing ratio in reflux decreased significantly with the increase of retrograde pressure (*p* < 0.001). Therefore, when the pressure increases to the actual value, the passing ratio of reflux will approach zero, indicating that the retractable valve can effectively prevent reflux.

FEA results show that the radial stiffness of the duodenal end of the anti-reflux stent is higher than that of the ordinary stent. In the clinic, the excessive radial stiffness of the stent will damage the bile duct and cause discomfort to patients. In this paper, the connection between the stent and the anti-reflux valve is located in the duodenum. Therefore, the increased radial stiffness of the duodenal end of the anti-reflux stent will not affect the function of the bile duct, but can reduce the negative impact of stent deformation on valve and stent migration (Minaga et al., 2016). The similar flexibility of anti-reflux stent and ordinary stent indicates that the valve attached to the end of the stent has no additional damage to the biliary duct and no additional operation difficulty of the stent through tortuous biliary duct (Mori and Saito 2005). In addition, the newly designed anti-reflux biliary stent could also adapt to the deformation of the biliary tract. These results are similar to common findings in reported (Hamada et al., 2015; Renno et al., 2019; Yuan et al., 2021).

It was reported that the clinical effect of the anti-reflux biliary stent was controversial. For instance, some studies pointed that anti-reflux biliary stents could significantly reduce the occurrence of duodenobiliary reflux and prolong the patency of stents, but the survival time of patients and the incidence of complications were not significantly different from the ordinary stent (Lee et al., 2016; Yuan et al., 2019). It has also been reported that the patency of some anti-reflux stents was shorter than that of ordinary stents (Vihervaara et al., 2017). The formation of biliary sludge was an important reason for the failure of the anti-reflux biliary stent (Lee et al., 2013; Hu et al., 2014). Sludge formation on the valve surface would cause the valve to harden and affect the antegrade flow of bile (Morita et al., 2018). In this paper, the results showed that this new anti-reflux biliary stent has the potential of preventing duodenobiliary reflux, improved radial mechanical performance, and similar flexibility compared with the ordinary stent without a valve. Further clinical study is needed to evaluate the safety and feasibility of the newly designed anti-reflux biliary stent.

There are also some limitations. Pathological bile presents properties of the non-Newtonian fluid (Li et al., 2008), but the medium used in this study was water. Biliary drainage is also dependent on stent diameter and length and bile viscosity (Coene et al., 1994). It is crucial to model bile as a non-Newtonian fluid and investigate the effect of a stent on bile flow. Recurrent biliary obstruction may be attributed to the dysfunction of anti-reflux valves (Hamada et al., 2019), and the pH environment of the duodenum may affect the morphology and function of valves (Kwon et al., 2018). In addition, the impact of stent implantation on the biliary system and potential complications should also be considered. *In vivo* evaluation is significant to verify the safety and effectiveness of the anti-reflux biliary stent.

## 8 Conclusion

This work has presented a novel anti-reflux biliary stent with a retractable bionic valve and has developed a 2D equivalent FSI model to estimate the mechanical performances of the anti-reflux biliary stent. The retrograde closure ratio of the retractable valve primarily relied on initial shear modulus of material (*p* < 0.05) but was not mainly dependent on thickness, pitch, and witch (*p* > 0.05). The optimized valve with a retrograde closing ratio of 95.89% has been proposed. The optimized anti-reflux biliary stent could ensure smooth bile drainage and prevent duodenobiliary reflux effectively. Compared with the ordinary stent, the optimized anti-reflux biliary stent had improved radial stiffness and similar flexibility. In addition, the 2D equivalent FSI model developed in this paper is significant for simplifying FSI simulation of other solids with large deformation.

## Data Availability

The original contributions presented in the study are included in the article/Supplementary Material, further inquiries can be directed to the corresponding author.
